# Prolonged treatment with pimelic *o*-aminobenzamide HDAC inhibitors ameliorates the disease phenotype of a Friedreich ataxia mouse model

**DOI:** 10.1016/j.nbd.2011.02.016

**Published:** 2011-06

**Authors:** Chiranjeevi Sandi, Ricardo Mouro Pinto, Sahar Al-Mahdawi, Vahid Ezzatizadeh, Glenn Barnes, Steve Jones, James R. Rusche, Joel M. Gottesfeld, Mark A. Pook

**Affiliations:** a Division of Biosciences, School of Health Sciences and Social Care, Brunel University, Uxbridge, UB8 3PH, UK; b Repligen Corporation, Waltham, MA, USA; c Department of Molecular Biology, The Scripps Research Institute, La Jolla, CA, USA

**Keywords:** Friedreich ataxia, FRDA, frataxin, Trinucleotide repeat, Transgenic mouse model, Histone deacetylase inhibitor, HDAC inhibitor

## Abstract

Friedreich ataxia (FRDA) is an inherited neurodegenerative disorder caused by GAA repeat expansion within the *FXN* gene, leading to epigenetic changes and heterochromatin-mediated gene silencing that result in a frataxin protein deficit. Histone deacetylase (HDAC) inhibitors, including pimelic *o*-aminobenzamide compounds 106, 109 and 136, have previously been shown to reverse *FXN* gene silencing in short-term studies of FRDA patient cells and a knock-in mouse model, but the functional consequences of such therapeutic intervention have thus far not been described. We have now investigated the long-term therapeutic effects of 106, 109 and 136 in our GAA repeat expansion mutation-containing YG8R FRDA mouse model. We show that there is no overt toxicity up to 5 months of treatment and there is amelioration of the FRDA-like disease phenotype. Thus, while the neurological deficits of this model are mild, 109 and 106 both produced an improvement of motor coordination, whereas 109 and 136 produced increased locomotor activity. All three compounds increased global histone H3 and H4 acetylation of brain tissue, but only 109 significantly increased acetylation of specific histone residues at the *FXN* locus. Effects on *FXN* mRNA expression in CNS tissues were modest, but 109 significantly increased frataxin protein expression in brain tissue. 109 also produced significant increases in brain aconitase enzyme activity, together with reduction of neuronal pathology of the dorsal root ganglia (DRG). Overall, these results support further assessment of HDAC inhibitors for treatment of Friedreich ataxia.

## Research Highlights

► We treated a Friedreich ataxia mouse model with histone deacetylase inhibitors. ► Improved motor coordination and locomotor activities. ► Increased histone acetylation at the *FXN* locus in brain. ► Increased frataxin protein, aconitase activity and decreased DRG pathology. ► 109 emerged as lead candidate drug for Friedreich ataxia therapy.

## Introduction

Friedreich ataxia (FRDA) is an autosomal recessive neurodegenerative disorder predominantly caused by a homozygous GAA repeat expansion mutation within intron 1 of the *FXN* gene (Campuzano et al., 1996). Normal individuals have 5–30 GAA repeat sequences, whereas affected individuals have from approximately 70 to more than 1000 GAA triplets. The effect of the GAA expansion mutation is to reduce the production of frataxin (Campuzano et al., 1997), a ubiquitously expressed mitochondrial protein that acts in iron–sulfur cluster and heme biosynthesis (Pandolfo and Pastore, 2009). Frataxin insufficiency leads to decreased activity of iron–sulfur cluster enzymes, such as aconitase and the mitochondrial respiratory chain complexes (Bradley et al., 2000), followed by mitochondrial iron accumulation and resultant cell death, with the primary sites of pathology being the large sensory neurons of the DRG and the dentate nucleus of the cerebellum (Koeppen et al., 2007; Koeppen et al., 2009). The outcome is progressive spinocerebellar neurodegeneration, causing symptoms of incoordination (“ataxia”), muscle weakness and sensory loss. There is also pathology of non-neuronal tissues, with cardiomyopathy a common secondary effect and diabetes found in 10% of FRDA patients (Schulz et al., 2009). Affected individuals are confined to a wheelchair within 20 years after the first appearance of symptoms, and most commonly die in early adulthood from the associated heart disease. Therefore, there is urgent need to develop an effective therapy for this lethal disorder.

Thus far, FRDA clinical trials using antioxidants and iron chelators have demonstrated some limited success at ameliorating secondary disease effects (Schulz et al., 2009). However, a more effective therapy may be achieved by targeting the more immediate effects of the GAA repeat expansion mutation to elevate deficient frataxin levels. Although the mechanisms by which the GAA repeat expansion leads to a frataxin deficit are currently not known, two non-exclusive hypotheses have been put forward. Firstly, it has been suggested that the GAA repeat expansion may adopt abnormal non-B DNA or DNA•RNA hybrid triplex structures that interfere with *FXN* gene transcription (Grabczyk et al., 2007; Wells, 2008). Secondly, there is evidence that GAA repeat expansions can produce a heterochromatin-mediated gene silencing effect (Saveliev et al., 2003), likely acting through epigenetic processes, such as DNA methylation and histone modifications. In support of this second hypothesis, recent studies of FRDA patient cells and tissues by ourselves and others have identified several GAA expansion-specific epigenetic changes (Al-Mahdawi et al., 2008; De Biase et al., 2009; Greene et al., 2007; Herman et al., 2006). Thus, GAA repeat expansions are associated with (i) increased DNA methylation in the region of *FXN* intron 1 immediately upstream of the GAA repeat (Al-Mahdawi et al., 2008; Greene et al., 2007); (ii) reduced H3K9 acetylation and increased H3K9 and H3K27 trimethylation at the *FXN* promoter (Al-Mahdawi et al., 2008; De Biase et al., 2009), and (iii) reduced acetylation of several H3 and H4 lysine residues together with increased H3K9 di- and trimethylation in both the upstream and downstream GAA regions (Al-Mahdawi et al., 2008; Greene et al., 2007; Herman et al., 2006). Therefore, there is ample evidence to propose reversal of *FXN* gene silencing by the use of epigenetic-modifying compounds as a potential therapy for FRDA.

HDAC inhibitors are a structurally diverse group of compounds that have been widely used in the treatment of various human cancers (Cang et al., 2009; Monneret, 2005; Prince et al., 2009). They are generally believed to act by increasing global histone acetylation and thereby reactivating epigenetically silenced genes, although HDAC inhibitors can also promote acetylation of non-histone proteins (Butler and Bates, 2006). Initial treatment of FRDA lymphoblastoid cells using a panel of commercially available HDAC inhibitors revealed that only the benzamide compound BML-210 (*N*^1^-(2-aminophenyl)-*N*^8^-phenyloctanediamide) produced a significant increase of *FXN* mRNA expression (Herman et al., 2006). Subsequent synthesis of analogs of BML-210 identified a pimelic *o*-aminobenzamide compound, 4b (*N*^1^-(2-aminophenyl)-*N*^7^-phenylheptanediamide), which was shown to act on FRDA primary lymphocytes to significantly increase acetylation of H3K14, H4K5 and H4K12 in the *FXN* upstream GAA region and to increase *FXN* mRNA levels by 2.5-fold (Herman et al., 2006). Further development of this family of benzamide HDAC inhibitors identified three other pimelic *o*-aminobenzamide compounds, 106 (*N*^1^-(2-aminophenyl)-*N*^7^-*p*-tolylheptanediamide), 136 (*N*-(6-(2-amino-4-fluorophenylamino)-6-oxyhexyl)-4-methylbenzamide) and 109 (*N*-(6-(2-aminophenylamino)-6-oxyhexyl)-4-methylbenzamide), which have each undergone investigations to determine pharmacokinetic properties, safety and efficacy in short-term treatments of FRDA patient cells and mice (Chou et al., 2008; Rai et al., 2010, 2008; Soragni et al., 2008; Xu et al., 2009). 106, 136 and 109 all act as slow-on, slow-off, tight binding inhibitors of class I HDACs, with a preference for HDAC3 (Chou et al., 2008; Rai et al., 2010; Xu et al., 2009). Furthermore, they all produce significant short-term increases in H4K5 acetylation and *FXN* mRNA and frataxin protein expression in FRDA primary lymphocytes and brain and heart tissues of FRDA KIKI mice (Rai et al., 2010, 2008).

We have established a GAA repeat expansion mutation-based mouse model of FRDA, designated YG8R, by cross breeding YG8 human genomic YAC transgenic mice that contain the entire *FXN* gene and expanded GAA repeats (Al-Mahdawi et al., 2004) with heterozygous *Fxn* knockout mice (Cossee et al., 2000). The resulting YG8R mice rescue the embryonic lethality of the *Fxn* homozygous knockout alleles by expressing only human frataxin from the GAA repeat-mutated *FXN* transgene in a mouse frataxin null background (Al-Mahdawi et al., 2006). The YG8R mice exhibit an FRDA-like molecular disease phenotype that includes intergenerational and somatic instability of the GAA repeat expansion mutation (Al-Mahdawi et al., 2004; Clark et al., 2007), together with increased DNA methylation, reduced histone H3 and H4 acetylation and increased di- and trimethylated H3K9 at the *FXN* upstream GAA region, when compared with Y47R control transgenic mice that contain the same human *FXN* YAC transgene but with normal-sized GAA repeats (Al-Mahdawi et al., 2008). Furthermore, YG8R frataxin mRNA levels in brain tissue are reduced to approximately 30% when compared with Y47R control transgenic mice (Al-Mahdawi et al., 2008). Although the YG8R mice do not show reduction of frataxin mRNA levels in brain tissue when compared with wild-type mice, frataxin protein levels are reduced to approximately 70% that of wild-type mice (Al-Mahdawi et al., 2006). YG8R mice are also likely to have impaired function of the human transgene-derived frataxin, resulting in deficits that are consistent with FRDA disease, including mildly impaired motor coordination ability, reduced aconitase enzyme activity and abnormal DRG neuronal pathology (Al-Mahdawi et al., 2006). Therefore, we consider YG8R to be a suitable mouse model in which to investigate potential FRDA therapies and we now report the effects of prolonged treatment with HDAC inhibitors 106, 136 and 109. Our results show that each compound is well tolerated during chronic subcutaneous dosing for up to 5 months. We detect improvements of behavioral, molecular, biochemical and histological measurements, particularly with 109, that are consistent with the compounds crossing the blood-brain barrier to ameliorate FRDA-like disease effects.

## Materials and methods

### Animal procedures

Genotyping of mice to identify human transgenic GAA sequences and endogenous mouse *Fxn* knockout alleles was performed as previously described (Pook et al., 2001). Mice were housed in conventional open cages with Litaspen Premium 8/20 bedding, paper wool nesting and standard fun tunnel environmental enrichment, with 13 h light, 11 h dark, 20-23 °C and 45–60% humidity. The mice were given a diet of SDS RM3 Expanded food pellets and standard drinking water. All procedures were carried out in accordance with the UK Home Office ‘Animals (Scientific Procedures) Act 1986.’

### Drug treatment

HDAC inhibitors 106 (*N*^1^-(2-aminophenyl)-*N*^7^-*p*-tolylheptanediamide), 136 (*N*-(6-(2-amino-4-fluorophenylamino)-6-oxyhexyl)-4-methylbenzamide) and 109 (*N*-(6-(2-aminophenylamino)-6-oxyhexyl)-4-methylbenzamide) were obtained from Repligen Corporation, Waltham, MA, USA, and were synthesized as previously described (Rai et al., 2010, 2008). 30 mg/ml 106 was prepared in a vehicle solution of 2.5% DMSO, 40% hydroxypropyl-β-cyclodextrin, 140 mM Na acetate pH5.2, and 10 mg/ml 136 and 109 were prepared in a vehicle solution of 20% glycerol, 20% PEG400, 20% propylene glycol, 5 mM Na acetate pH 5.2. Mice were given subcutaneous injections of 150 mg/kg 106 three times per week for 4.5 months, or 50 mg/kg 136 or 100 mg/kg 109 five times per week for 5 months, followed by culling for tissue collection 24 h after the final injection.

### Behavioral assessments

Rotarod performances of YG8R and C57BL/6J wild-type mice were assessed using a Ugo Basile 7650 accelerating rotarod treadmill and open field locomotor activity was assessed using a gridded perspex box in light room conditions, as previously described (Al-Mahdawi et al., 2006). Ambulatory distance and vertical counts were measured over 2 min periods in the dark and repeated four times for each mouse using a beam-breaker activity monitor (Medical Devices, Inc.). For final analysis, the fold change in mean performance time for each group of mice was calculated and compared with the original mean performance time.

### Quantitative reverse transcriptase PCR

Total RNA was isolated from snap frozen tissues by homogenization with Trizol (Invitrogen) and cDNA was then prepared by using AMV reverse transcriptase (Invitrogen) with oligo-dT primers. Levels of human transgenic *FXN* or endogenous *Fxn* mRNA expression were assessed by quantitative RT-PCR using an ABI7900 sequencer and SYBR® Green (Applied Biosystems) with the following primers that equally amplify human and mouse sequences: FRT-I 5'-TTGAAGACCTTGCAGACAAG-3' and RRT-II 5'-AGCCAGATTTGCTTGTTTGG-3'. Mouse *Gapdh* RT-PCR primers used for normalization were as follows: GapdhmF 5'-ACCCAGAAGACTGTGGATGG-3' and GapdhmR 5'-GGATGCAGGGATGATGTTCT-3'.

*Gapdh* mRNA expression was similarly quantified using GapdhmF and GapdhmR primers and using mouse *Rer1* RT-PCR primers for normalization, as follows: RER1-F: 5'-CCACCTAAACCTTTTCATTGCG-3' and RER1-R: 5'-TTTGTAGCTGCGTGCCAAAAT-3'.

Reactions were carried out in triplicate for each biological sample (*n* = 4).

### Western blot analysis

Protein lysates were obtained from snap frozen brain tissues, and frataxin Western blot analysis was carried out as previously described (Pook et al., 2001), using an anti-frataxin rabbit polyclonal antibody (Santa Cruz 25820). Histone H3 and H4 acetylation levels were determined by Western blot analysis using anti-acetyl-Histone H3 (Upstate 06-599) and anti-acetyl-Histone H4 (Upstate 06-866) antibodies, respectively. In all cases, normalization was carried out using a rat anti-α-tubulin monoclonal antibody (Abcam AB6160). Densitometry was carried out using UN-SCAN-IT software (Silk Scientific Corporation) and in all cases (*n* = 4).

### ChIP analysis

ChIP analysis was carried out by initial cross-linking of DNA and protein by formaldehyde treatment of homogenized frozen tissue samples. DNA was then sheared by sonication, followed by immunoprecipitation with commercially available anti-acetylated histone H3 and H4 antibodies: H3K9ac, H4K5ac, and H4K12ac (Upstate). For each experiment, normal rabbit serum (SIGMA) was used as a minus antibody immunoprecipitation control. After reversal of cross-linking, quantitative PCR amplification of the resultant co-immunoprecipitated DNA was carried out with SYBR® Green in an ABI7900 sequencer (Applied Biosystems) using three sets of *FXN* primers (Pro, Up and Down) and mouse *Gapdh* primers, as previously described (Al-Mahdawi et al., 2008; Herman et al., 2006). Each tissue sample was subjected to two independent ChIP procedures, followed by triplicate quantitative PCR analysis.

### Aconitase activity assay

Aconitase activities were determined by homogenization of mouse brain tissues on ice at 10% w/v in CellLytic MT Mammalian Tissue Lysis/Extraction buffer (SIGMA, C3228), followed by centrifugation at 800 × *g* for 10 min at 4 °C. Tissue lysates (50 μl) were then added to 200 μl of substrate mix (50 mM Tris/HCl pH 7.4, 0.4 mM NADP, 5 mM Na citrate, 0.6 mM MgCl_2_, 0.1% (v/v) Triton X-100 and 1U isocitrate dehydrogenase) and the reactions were incubated at 37 °C for 15 min, followed by spectrophotometric absorbance measurements every minute for 15 min at 340 nm 37 °C to determine the reaction slope. Aconitase activities of mouse brain tissues were then normalized to citrate synthase activities, which were determined using a citrate synthase assay kit (SIGMA, CS0720).

### DRG histology

Histological preparations of mouse DRG were carried out by dissection of paraformaldehyde-fixed intact lumbar vertebrae, followed by decalcification treatment in Hillman and Lee's EDTA daily for 5 days. Tissues were then embedded in paraffin wax, sectioned by standard methods, deparaffinized with IMS and Histoclear (National Diagnostics) and slides were stained with hematoxylin and eosin.

### Statistical analysis

Body weight, rotarod performance, locomotor activity, ambulatory distance and vertical counts beam-breaker data were analyzed by two-way ANOVA for genotype (YG8R or wild-type) and time (age of mice) to detect disease-like effects and for treatment (HDAC inhibitor or vehicle) and time (age of mice) to detect drug effects. All other data were analyzed by the Student's *t* test, with a significance value set at *p* < 0.05.

## Results

### HDAC inhibitors 106, 136 and 109 are well tolerated in chronic dosing of mice

HDAC inhibitors 106, 136 and 109 have previously been administered subcutaneously to a separate GAA knock-in mouse model (KIKI mice, where GAA repeats have been introduced into the mouse frataxin gene) daily for a period of 3 days, without any toxic effects (Rai et al., 2010, 2008). We wanted to determine if this lack of acute toxicity could be maintained throughout long-term dosing regimes. We initially treated one group of fifteen 3- to 4-month-old YG8R mice with 150 mg/kg 106 by subcutaneous injection 3 times per week for a period of 4.5 months, together with an age- and sex-matched YG8R control group treated with vehicle only. We subsequently treated three groups of 20 age- and sex-matched 3- to 4-month-old YG8R mice with either 50 mg/kg 136, 100 mg/kg 109 or vehicle-only by subcutaneous injection five times per week for a period of 5 months. At the same time, we similarly treated three groups of 10 age-and sex-matched 3- to 4-month-old wild-type (C57BL/6 J) mice (YG8R mice are in a C57BL/6 J genetic background and Y47R control transgenic mice were not available for this study) with either 50 mg/kg 136, 100 mg/kg 109 or vehicle-only by subcutaneous injection five times per week for a period of 5 months.

The long-term administrations of 106, 136 and 109 were well tolerated and no toxicity was generally observed in the mice throughout the entire period of study. However, one 136-treated YG8R mouse died from an unknown cause after 4 months and one 109-treated wild-type mouse showed a failure to thrive after 2 months and was therefore culled. Analysis of body weight over time by two-way ANOVA detected a non-significant (*F* = 2.9, *p* = 0.086) reduction in the weight gain of YG8R mice compared with wild-type mice. This mild detrimental phenotype remained unaffected by prolonged treatment with 109 (*F* = 1.28, *p* = 0.25), but treatment with 106 and 136 resulted in significant further reductions in weight gain (*F* = 13.5, *p* < 0.001 and *F* = 5.0, *p* < 0.05, respectively) (Fig. S1), indicating that 109 is the best tolerated of the three compounds under the dosing conditions tested.

### 106 and 109 improve motor coordination of YG8R FRDA mice

**Fig. 1:**
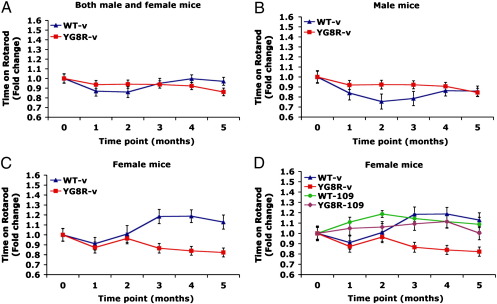
Motor coordination analysis. Relative rotarod performances are shown for vehicle-treated YG8R FRDA mice (red) and wild-type (WT) mice (blue) as fold-changes over a 5-month time period. (A) Combined male and female values (YG8R *n* = 20, WT *n* = 10), (B) male values only (YG8R *n* = 10, WT *n* = 5), and (C) female values only (YG8R *n* = 10, WT *n* = 5) are shown. (D) Values for 109-treated YG8R FRDA mice (purple) and wild-type (WT) mice (green) are shown together with vehicle-treated groups (YG8R *n* = 10, WT *n* = 5). Error bars represent s.e.m.

Motor coordination was assessed by change in performance on a rotarod treadmill at monthly time points throughout the treatment periods. An initial comparison of vehicle-treated YG8R and wild-type mice revealed a gender-specific phenotype, which has not previously been investigated or reported. Thus, no significant difference in rotarod performance was noted when both male and female values were taken together (Fig. 1A) or when male values were considered alone (Fig. 1B). However, analysis of female values alone revealed a significantly reduced rotarod performance (*F* = 35.9, *p* < 0.001) in YG8R mice (Fig. 1C). With this in mind, only the rotarod performances of female mice were taken into account when determining any effect of the HDAC inhibitors on motor coordination. Both 106 and 109 treatments were found to improve the rotarod performances of YG8R female mice (*F* = 44.2, *p* < 0.001 and *F* = 20.0, *p* < 0.001, respectively) (Fig. 1D and Fig. S1D), while 136 treatment showed no effect. Furthermore, the rotarod improvement achieved by 109 treatment appeared to be specific for YG8R mice, since no effect was seen with 109 treatment of wild-type female mice (Fig. 1D).

### 136 and 109 improve locomotor activity of YG8R FRDA mice

**Fig. 2:**
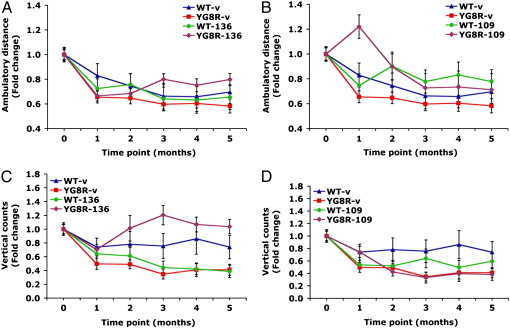
Beam-breaker behavior analysis. Relative ambulatory distance (A, B) and vertical counts (C, D) are shown as fold-changes for vehicle-treated groups of YG8R FRDA mice (red) and wild-type (WT) mice (blue) over a 5-month time period. (A) and (C) show 136-treated YG8R FRDA mice (purple) and wild-type (WT) mice (green). (B) and (D) show 109-treated YG8R FRDA mice (purple) and wild-type (WT) mice (green). (YG8R *n* = 20, WT *n* = 10). Error bars represent s.e.m.

Several parameters were used to measure locomotor activity of mice. However, unlike the analysis of motor coordination, no gender-specific YG8R phenotype was observed and therefore the combined male and female data are presented. Firstly, the open field activity of 106- and 136-treated YG8R mice compared with vehicle-treated YG8R mice was assessed by determining the distance moved within a gridded perspex box under normal light conditions, as previously described (Al-Mahdawi et al., 2006). 106 treatment had no effect on activity, whereas 136 treatment significantly increased activity in YG8R mice (*F* = 17.7, *p* < 0.001) (Fig. S1E). Secondly, for the study of 136- and 109-treated YG8R and wild-type mice, beam-breaker activity monitor apparatus was used to measure (i) the total horizontal distance moved (‘ambulatory distance’) and (ii) a count of the vertical movements due to rearing on hind limbs (‘vertical counts’) within a 2-min period under dark conditions. Initial analysis by two-way ANOVA detected a significant ambulatory distance disease-like phenotype of YG8R mice compared with wild-type mice (*F* = 6.0, *p* < 0.05). Subsequent treatments with 136 and 109 both produced significant increases in the ambulatory distance of YG8R mice towards wild-type values (*F* = 13.6, *p* < 0.001 and *F* = 22.3, *p* < 0.001, respectively) (Fig. 2A and B). These increases were specific for YG8R mice, since 136 produced a non-significant decrease (*F* = 0.34, *p* = 0.56), while 109 produced a non-significant increase (*F* = 1.92, *p* = 0.167), in the ambulatory distance of wild-type mice (Fig. 2A and B). Two-way ANOVA analysis also detected a significant vertical counts disease-like phenotype of YG8R mice compared with wild-type mice (*F* = 24.5, *p* < 0.001). Only 136 treatment caused a significant increase in the vertical counts of YG8R mice compared to vehicle-treated controls (*F* = 67.4, *p* < 0.001) (Fig. 2C), and this effect was specific for YG8R, because the values were significantly decreased in 136-treated wild-type mice (*F* = 8.6, *p* < 0.01). 109 treatment was shown to have no effect (*F* = 1.7, *p* = 0.19) (Fig. 2D).

### 106, 136 and 109 increase general histone acetylation levels in the brain

**Fig. 3:**
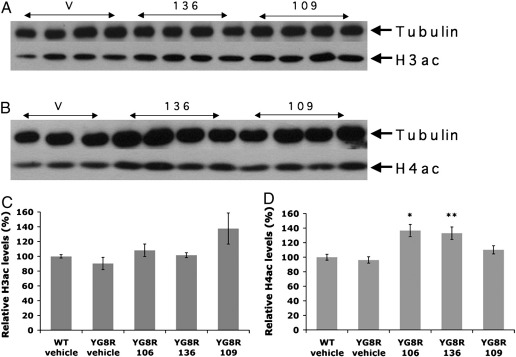
General H3 and H4 acetylation levels in YG8R brain tissues. Western blots showing (A) acetylated H3 histones (H3ac) and (B) acetylated H4 histones (H4ac) from vehicle (v)-, 136- and 109-treated YG8R mouse brain samples, together with α-tubulin controls. Densitometry calculations of acetylated H3 histones (C) and acetylated H4 histones (D) are shown for vehicle-treated wild-type (WT) mice, and vehicle-, 106-, 136- and 109-treated YG8R mouse brain samples. Values are shown relative to the vehicle-treated WT mouse value. In all cases, *n* = 4 and error bars represent s.e.m. *p < 0.05, **p < 0.01.

To examine the effects of prolonged dosing of 106, 136 and 109 on general histone acetylation within the brain tissue of YG8R and wild-type mice, acetylated H3 and acetylated H4 levels were determined by Western blotting analysis (Fig. 3). No differences in either acetylated H3 or acetylated H4 levels were detected between wild-type and YG8R mice. However, increases in both acetylated H3 and acetylated H4 levels were detected in YG8R mice treated with all three HDAC inhibitors compared to vehicle-treated controls, and similar increases were seen in HDAC inhibitor-treated wild-type mice. Compound 109 exhibited a more pronounced effect on acetylated H3 levels, which were increased by 1.5-fold, although not to statistical significance (Fig. 3C). In contrast, 106 and 136 had more prominent effects on acetylated H4 levels, each producing significant 1.4-fold increases (*p* < 0.05 and *p* < 0.01, respectively) (Fig. 3D).

### 109 increases acetylated H3K9, H4K5 and H4K12 within the FXN transgene and the endogenous mouse gapdh gene in YG8R brain tissue

**Fig. 4:**
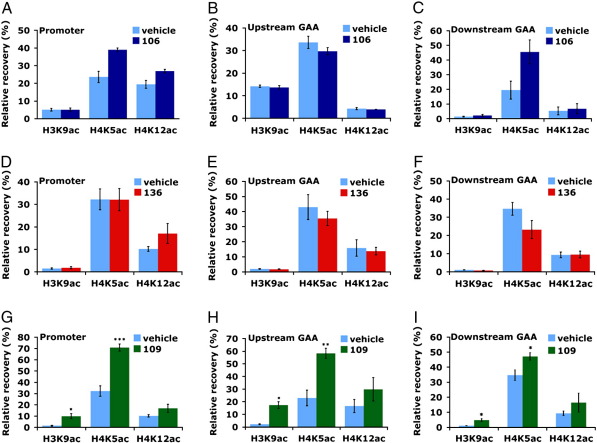
ChIP analysis of acetylated H3K9, H4K5 and H4K12 residues within the *FXN* locus of HDAC inhibitor- and vehicle-treated YG8R brain tissues. ChIP quantitative PCR results for H3K9ac, H4K5ac and H4K12ac residues within the transgenic *FXN* promoter (A, D, G), upstream GAA region (B, E, H) and downstream GAA region (C, F, I) of mouse brain tissue are represented as relative recovery (%), which was calculated as the amount of immunoprecipitated DNA compared to input DNA, subtracting the –Ab background value. The effects of treatment with 106 (A–C, dark blue), 136 (D–F, red) and 109 (G–I, green) are shown in comparison to vehicle-treatments (light blue). *n* = 3–6 and error bars represent s.e.m. *p < 0.05, **p < 0.01, ***p < 0.001.

We have previously detected comparatively reduced levels of acetylated H3 and H4 histone residues in brain tissue of expanded GAA repeat YG8 transgenic mice compared with normal-sized GAA repeat Y47 transgenic mice by performing chromatin immunoprecipitation (ChIP) analysis at three regions of the *FXN* gene: the promoter, upstream of the GAA repeat and downstream of the GAA repeat (Al-Mahdawi et al., 2008). Therefore, to investigate HDAC inhibitor effects after prolonged treatment of YG8R mice, we performed ChIP analysis of acetylated H3K9, H4K5 and H4K12 residues at the same three *FXN* regions, comparing results from HDAC inhibitor-treated and vehicle-treated YG8R mouse brain tissues. We chose to examine H3K9 and H4K12 because these residues previously showed the greatest decreases in acetylation in YG8 transgenic mice, and H4K5 was chosen because this residue is known to be the most preferentially modified by HDAC3, the principle HDAC target of the pimelic *o*-aminobenzamide HDAC inhibitors (Chou et al., 2008; Xu et al., 2009). Treatment with 106 produced little effect, although non-significant 1.6-fold and 2.3-fold increases of acetylated H4K5 were observed in the *FXN* promoter and downstream GAA regions, respectively (Fig. 4A–C). Similarly, treatment with 136 showed no effect (Fig. 4D–F). However, 109 treatment produced striking increases in each of the three acetylated H3K9, H4K5 and H4K12 residues in all three regions of the *FXN* locus, with the most statistically significant increases found at H3K9 and H4K5 residues. The largest increases, measured by fold-change, were detected for the acetylated H3K9 residue, with 6-fold, 8-fold and 4.5-fold increases at the promoter, upstream GAA and downstream GAA regions, respectively (Fig. 4G–I). However, the most statistically significant increases were observed for the acetylated H4K5 residue at the promoter and upstream GAA regions (Fig. 4G and H). At the same time, as a control experiment, we investigated 109 effects on acetylation of H3K9, H4K5 and H4K12 in the endogenous mouse *Gapdh* gene, using the same mouse brain tissues. As with the *FXN* gene, 109 produced increases in all three acetylated histone residues, with significant 8-fold and 1.8-fold increases in H3K9 and H4K5, respectively (Fig. S2A).

### FXN mRNA levels in CNS tissues are not significantly altered by prolonged 106, 136 or 109 treatment

To investigate if the increased acetylation that we found in *FXN* histone residues was reflected by changes of *FXN* transcription, we determined *FXN* mRNA levels in brain and other CNS tissues by quantitative RT-PCR, comparing HDAC inhibitor-treated and vehicle-treated YG8R and wild-type samples. Surprisingly, no substantial increases in *FXN* expression were detected following any of the three HDAC inhibitor treatments (Figs. S3 and S4). Some modest increases of *FXN* expression were detected, particularly in spinal cord samples, but none of the changes were statistically significant. However, the relative *FXN* expression levels in 136- and 109-treated tissues, compared with vehicle-treated tissues, were consistently higher in YG8R mice than in WT mice, suggesting some element of specificity towards increasing *FXN* expression in the transgenic FRDA mouse model (Fig. S3). As our ChIP experiments had also revealed a significant effect of 109 on acetylated H3 and H4 histone residues within the endogenous *Gapdh* gene, we also assessed brain *Gapdh* mRNA expression levels but detected no significant changes (Fig. S2B).

### 109 increases frataxin protein expression in the brain of YG8R FRDA mice

**Fig. 5:**
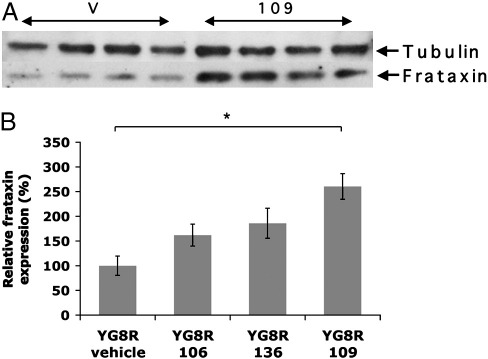
Frataxin protein levels in YG8R brain tissues. (A) Representative Western blot showing frataxin protein expression, together with α-tubulin controls, from vehicle (v)- and 109-treated YG8R mouse brain tissues. (B) Densitometry calculations of frataxin levels are shown for vehicle-, 106-, 136- and 109-treated YG8R mouse brain samples. Values are shown relative to the vehicle-treated YG8R mouse value. In all cases *n* = 4 and error bars represent s.e.m. *p < 0.05.

**Fig. 6:**
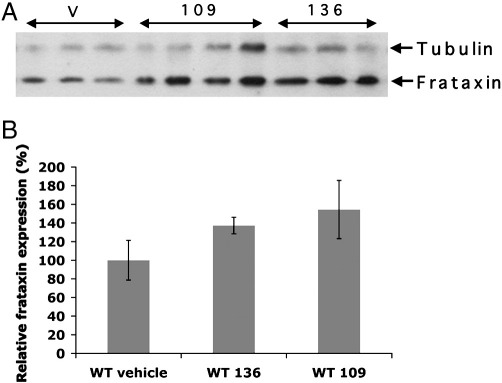
Frataxin protein levels in wild-type mouse brain tissues. (A) Western blot showing frataxin protein expression, together with α-tubulin controls, from vehicle (v)-, 109- and 136-treated wild-type mouse brain tissues. (B) Densitometry calculations of frataxin levels are shown for vehicle-, 136- and 109-treated wild-type (WT) mouse brain samples. Values are shown relative to the vehicle-treated WT mouse value and increases were not statistically significant. In all cases *n* = 4 and error bars represent s.e.m.

The same brain tissue samples that had been used to determine *FXN* expression levels were also used to assess comparative levels of frataxin protein expression by Western blotting analysis (Fig. 5A). Modest 1.6-fold and 2-fold increases were seen in 106- and 136-treated YG8R mice, respectively, compared with vehicle-treated YG8R mice. However, 109-treated YG8R mice revealed a much more significant 2.6-fold increase in frataxin expression when compared with vehicle-treated YG8R mice (*p* < 0.05) (Fig. 5B). 1.3-fold and 1.5-fold increases in endogenous mouse frataxin levels were also seen in 136- and 109-treated wild-type mice, respectively, suggesting a minor generalized frataxin-increasing effect of these drugs, although the increases were not statistically significant (Fig. 6).

### 109 increases aconitase enzyme activity within YG8R mouse brain tissue

**Fig. 7:**
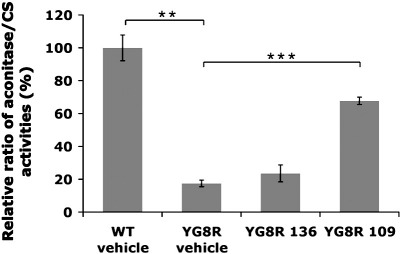
Aconitase activity of YG8R brain tissues. Aconitase activities for vehicle-treated wild-type (WT) mice, and vehicle-, 136- and 109-treated YG8R mouse brain samples are shown as ratios of the corresponding citrate synthase activity and relative to the vehicle-treated WT mouse value set at 100%. *n* = 3–8 and error bars represent s.e.m. **p < 0.01, ***p < 0.001.

A consistently observed effect of the frataxin deficit in FRDA patients, FRDA mouse models and yeast models is a reduction in activity of the iron–sulfur cluster containing enzyme aconitase (Bradley et al., 2000; Puccio et al., 2001; Rotig et al., 1997). We have also previously detected reduced levels of aconitase activity in heart tissue from our YG8R mice (Al-Mahdawi et al., 2006). Therefore, we were interested to determine whether aconitase activity was also reduced in YG8R brain tissue and whether such activity could be influenced by HDAC inhibitor treatment. Our analysis shows that there is indeed a significant reduction in YG8R brain aconitase activity to 17% of wild-type levels (*p* < 0.001) (Fig. 7). This reduced aconitase activity was subsequently increased 1.4-fold by treatment with 136. However, a much more significant 4-fold increase occurred with 109 treatment (*p* < 0.001), returning the aconitase activity substantially towards wild-type levels.

### 109 ameliorates DRG neuronal pathology

**Fig. 8:**
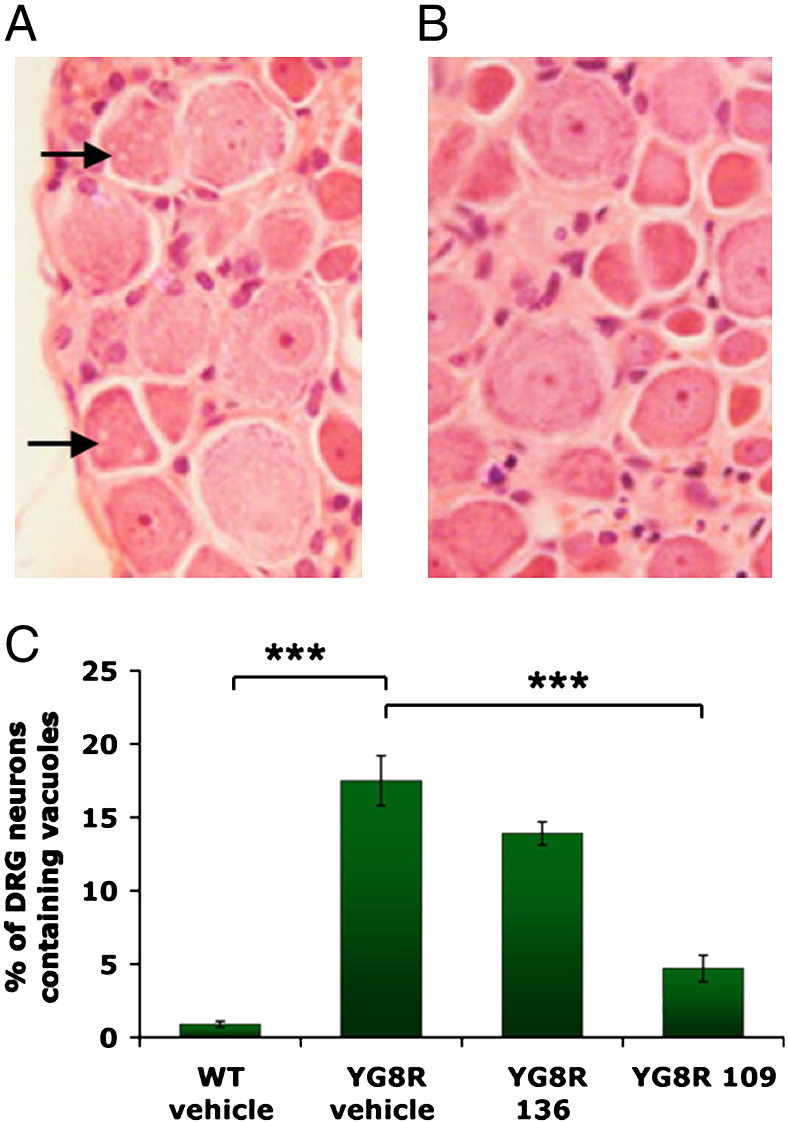
DRG neuronal pathology: (A) Representative H&E-stained histology sections of lumber DRG from (A) vehicle-treated and (B) 109-treated YG8R mice. Vacuolar pathology of neuronal cell bodies, which is present only in the vehicle-treated section (A), is indicated by the arrows. Original magnificatio*n* = 400×. (C) Graphic representation showing the degree of DRG histopathology in vehicle-treated wild-type (WT) and vehicle-, 136- and 109-treated YG8R mice, calculated as the percentage of neurons containing vacuoles compared to the total number of neurons. In all cases *n* = 16 and error bars represent s.e.m. ***p < 0.001.

We initially confirmed the presence of vacuolar pathology within the large neuronal cell bodies of lumbar DRG as a disease phenotype within the vehicle-treated YG8R mice, as we have previously described (Al-Mahdawi et al., 2006) (Fig. 8A). Quantification of this vacuolar pathology was then performed by calculating the number of vacuole-containing neuronal cells as a percentage of total neuronal cells within the DRG sections, resulting in a value of 17.5% for YG8R mice, compared with only 0.9% for wild-type mice. 136 treatment was found to produce a small reduction in the level of DRG pathology, with the vacuole count reaching a value of 13.9% (*p* = 0.06) (Fig. 8C). However, 109 treatment produced a much more significant reduction in DRG pathology (Fig. 8B), with the vacuole count reaching a value of 4.7% (*p* < 0.001) (Fig. 8C), thus indicating substantial amelioration of the disease-like phenotype.

## Discussion

The recent understanding that FRDA disease pathogenesis involves GAA expansion-induced histone deacetylation of the *FXN* gene has now led to the proposed use of HDAC inhibitors for FRDA therapy (Gottesfeld and Pandolfo, 2009). Initial short-term dosing studies with the pimelic *o*-aminobenzamide HDAC inhibitors 106, 109 and 136 have demonstrated efficacy at up-regulating frataxin expression in FRDA cells and KIKI mouse model tissues, while exhibiting minimal toxicity (Chou et al., 2008; Herman et al., 2006; Rai et al., 2010, 2008; Soragni et al., 2008). In this present study, we investigated the chronic dosing effects of 106, 109 and 136 in our YG8R FRDA mouse model. We report for the first time that prolonged subcutaneous administrations of 106, 109 and 136 are all well tolerated in mice at the tested doses, concurring with a recent long-term study of the related HDAC inhibitor 4b, which demonstrated amelioration of the Huntington disease phenotype in R6/2 mice without any discernable toxicity (Thomas et al., 2008). Furthermore, we report treatment-induced increases of acetylated histones and frataxin expression levels in YG8R mouse brain, demonstrating that 106, 109 and 136 can each cross the blood–brain barrier to exert HDAC inhibitory effects, in agreement with previous short-term KIKI mouse studies (Rai et al., 2010, 2008).

The HDAC inhibitor treatments did not elicit any dramatic changes in the behavior deficits of YG8R mice, but modest changes were moved in the improved direction by drug treatment, including improved motor coordination with 106 and 109 and increased locomotor activity with 109 and 136. Such differential behavioral effects may be due to differences in HDAC inhibitor potency, specificity, tissue distribution and effectiveness at crossing the blood–brain barrier. For example, 109, which elicited improvements in both behavioral measurements, has been reported to exhibit selectivity for HDAC3, but retains the most potency for HDAC1 and HDAC2 among these three pimelic *o*-aminobenzamide compounds (Rai et al., 2010).

Our results of the treatment effects on histone acetylation at the *FXN* locus by ChIP analysis of brain tissue are in general agreement with previous studies but substantially extend these studies and, as a consequence, provide further evidence for the greater potency of 109 compared with 106 or 136. Thus, we have shown that 109 treatment significantly increases acetylation of H4K5 at the *FXN* upstream GAA region, while 136 treatment has little effect, as similarly described for studies of FRDA patient PBMCs and KIKI mouse brain tissues (Rai et al., 2010). However, we did not detect any 106-induced increase in acetylation of H4K5 at the *FXN* upstream GAA region, contrary to the 106 effect reported for the KIKI mouse (Rai et al., 2008). This discrepancy is most likely due to the different frataxin intron 1 sequences, including gene regulatory sequences, which have been studied in the two mouse models; the YG8R mouse has human *FXN* transgenic sequence, while the KIKI mouse has murine *Fxn* sequence. Our study is the first study to report any treatment effects of 106, 109 or 136 on the *FXN* upstream GAA region acetylation status of residues H3K9 and H4K12; the two histone residues that show the greatest deacetylation in FRDA disease (Al-Mahdawi et al., 2008; Herman et al., 2006; Soragni et al., 2008). Significant increases in acetylation of H3K9 were identified only with 109 treatment. In contrast, 106 and 136 had no effect, as previously described for the related HDAC inhibitor 4b that similarly did not induce any H3K9 acetylation changes in the upstream GAA region of human FRDA lymphoblastoid cells but rather preferentially increase acetylation of H3K14 and H4K5 (Herman et al., 2006). Our study also detected treatment effects of HDAC inhibitors on histone acetylation at the *FXN* promoter and downstream GAA regions. We identified modest 106-induced increases in acetylation of H4K5 in both regions and modest increases in acetylation of H4K12 in the *FXN* promoter after treatment with all three compounds. However, the most significant changes that we detected were 109-induced increases in H3K9 and H4K5 acetylation.

Despite the marked 109-induced increases that we detected in acetylated H3 and H4 residues at the *FXN* locus in brain tissues of YG8R mice, we did not find any corresponding increase in brain *FXN* mRNA expression, but we did detect a significant 2.6-fold increase in frataxin protein expression. Differential timings of frataxin mRNA and protein expression could well account for this apparent discrepancy or possibly dose levels and dose frequency did not achieve maximal exposure and efficacy for each compound. Moreover, we cannot rule out the possibility of 109-induced post-transcriptional effects on frataxin expression, such as increased frataxin protein stability, as suggested by our detection of small increases in endogenous mouse frataxin in HDAC inhibitor-treated wild-type mice as well as the more significant increase of human transgenic frataxin in the 109-treated YG8R mice. HDAC inhibitors have been shown to change the transcription of many genes (Drummond et al., 2005; Rai et al., 2008; Reid et al., 2005; Thomas et al., 2008) and some gene expression changes may result in enhanced protein stability, as described in one recent study (Garbes et al., 2009). A similar finding to ours has also been reported for the potential FRDA therapeutic compound rhuEPO, where treatment of primary FRDA fibroblasts produced up to 2-fold increases in frataxin protein levels without any changes in *FXN* mRNA expression (Acquaviva et al., 2008). The authors of this study similarly hypothesized that rhuEPO may stabilize frataxin protein by an as yet unknown mechanism. More extensive studies will therefore be necessary to determine the mechanism(s) whereby the pimelic *o*-aminobenzamide HDAC inhibitors increase frataxin protein levels. Interestingly, one recent study has identified lysine residue 147 to play a critical role in frataxin ubiquitination and degradation (Rufini et al., 2011). Therefore, if acetylation of lysine 147 also occurs as a competing post-translational modification of frataxin, or indeed if any of the other 12 frataxin lysine residues prove to be acetylated, HDAC inhibitors could potentially exert an effect of promoting frataxin protein stability.

Aconitase is an Fe–S-containing enzyme that exists in both mitochondrial and cytosolic forms, and a deficit in aconitase activity has previously been identified as a major effect of decreased frataxin in FRDA patients, mouse models and yeast models (Al-Mahdawi et al., 2006; Bradley et al., 2000; Puccio et al., 2001; Rotig et al., 1997). Thus, aconitase activity can be considered a suitable biomarker of FRDA disease. The substantial deficit of aconitase activity that we identified in the brain tissue of our YG8R mouse (14% of wild-type) is similar to deficits that have been reported for human tissues (Bradley et al., 2000), but perhaps more substantial than may have been expected considering the comparatively mild degree of brain frataxin protein reduction in this mouse model (70% of wild-type) (Al-Mahdawi et al., 2006). However, it is very likely that the transgenic human frataxin protein produced within the YG8R mouse will not function as effectively as endogenous mouse frataxin protein due to amino acid sequence differences, thereby compounding the frataxin deficit and hence the aconitase deficit. Most interestingly, after treatment with HDAC inhibitors, the best improvement in aconitase activity was found to be with 109, the HDAC inhibitor that produced the most significant changes in histone acetylation, together with the largest subsequent increases in frataxin expression.

Degeneration of the large sensory neurons within the DRG has long been known as a prominent feature of FRDA disease (Koeppen et al., 2009), and more recently the formation of pathological vacuoles within these particular cells has also been reported in several FRDA mouse models (Al-Mahdawi et al., 2006; Puccio et al., 2001; Simon et al., 2004). Using the occurrence of DRG vacuoles as a marker of FRDA disease pathology in our YG8R mice, we have shown that, as with restoration of the aconitase deficit, the HDAC inhibitor that produced the most significant improvement in DRG neuronal cell pathology is 109, the HDAC inhibitor that produced the most significant increase in frataxin expression. This suggests that 109 may be exerting beneficial effects on both aconitase activity and neuronal pathology as a direct consequence of its ability to increase frataxin protein expression. However, it is also possible that some of the amelioration of neuronal pathology is due to an additional more general neuroprotective effect of 109, as previously described for the effect of HDAC inhibitor 4b on the R6/2 HD mouse model (Thomas et al., 2008). Therefore, future microarray gene expression analysis of 109-treated mouse tissue would be useful to determine potential upregulation of neuroprotective genes by 109 treatment.

## Conclusions

We have shown that prolonged treatment with each of the three HDAC inhibitors, 106, 136 and 109 can ameliorate FRDA disease-like effects to some extent, and therefore our studies are supportive of advancing HDAC inhibitors for treating FRDA. Furthermore, 109 clearly emerges as a lead drug candidate due to its ability to increase brain frataxin protein and aconitase activity and reduce DRG neuronal pathology in our FRDA mouse model. These beneficial effects are also likely to account for the modest improvements in motor coordination performance and locomotor activity that we observed with 109 treatment. However, the behavioral deficits of the YG8R mouse model are still rather mild, and therefore, by exploiting the unstable nature of the GAA repeat mutation, we are currently developing a larger GAA repeat-based FRDA mouse model with more substantial reduction in *FXN* expression and a more severe behavioral phenotype that would be beneficial to future drug testing. It may also be useful to investigate the more global effects of prolonged 109 treatment by performing microarray gene expression profiling of treated YG8R mice, in a similar manner to studies of the related HDAC inhibitors 4b and 106 that have each identified expression changes in only a limited number of genes (Rai et al., 2008; Thomas et al., 2008). Several HDAC inhibitors have now been evaluated for the treatment of a variety of genetic disorders, such as spinal muscular atrophy (SMA) (Garbes et al., 2009; Hahnen et al., 2006; Kernochan et al., 2005; Riessland et al., 2006), Huntington disease (Butler and Bates, 2006; Hockly et al., 2003; Thomas et al., 2008), cardiac hypertrophy (Antos et al., 2003) and cystic fibrosis (Hutt et al., 2010). To add to this list, we have now shown that the pimelic *o*-aminobenzamide compound 109 is safe and effective at up-regulating frataxin expression in prolonged treatment of an FRDA mouse model. These studies support continued evaluation of HDAC inhibitors to treat Friedreich ataxia.

The following are the supplementary materials related to this article.

Fig. S1 Weight and behavioral analysis. (A–C) Fold changes in body weights are shown for vehicle-treated YG8R (red) and wild-type (WT) (blue) mice and HDAC inhibitor-treated YG8R (purple) and wild-type (WT) (green) mice over a 5-month time period. (A) 106 (*n* = 15), (B) 136 (YG8R *n* = 20, WT *n* = 10) and (C) 109 (YG8R *n* = 20, WT *n* = 10). (D) Relative rotarod performances of 106-treated (blue) and vehicle-treated (red) YG8R FRDA mice (*n* = 15). (E) Relative open field locomotor activities of 136-treated (blue) and vehicle-treated (red) groups of YG8R FRDA mice (*n* = 20). Error bars represent s.e.m.

Fig. S2 *Gapdh* ChIP and mRNA expression levels. (A) ChIP analysis of acetylated H3K9, H4K5 and H4K12 residues within the endogenous mouse *Gapdh* gene of 109- and vehicle-treated YG8R mouse brain tissues are represented as relative recovery (%), which was calculated as the amount of immunoprecipitated DNA compared with input DNA, subtracting the –Ab background value (*n* = 6). (B) Relative *Gapdh* mRNA expression of YG8R mouse brain tissue, compared with a vehicle-treated control value set at 100% (*n* = 3). Error bars represent s.e.m. *p < 0.05, ***p < 0.001.

Fig. S3 Relative *FXN* mRNA levels in HDAC inhibitor-treated YG8R and wild-type brain tissues. Relative *FXN* mRNA expression levels in the brain, spinal cord, DRG and cerebellum of YG8R mice (A, B) and wild-type mice (C, D) are shown after treatment with 136 (A and C) and 109 (B and D), compared with corresponding vehicle-treated controls set at 100%. In all cases *n* = 4.

Fig. S4 *FXN* mRNA levels in 106-treated brain. Relative *FXN* mRNA expression levels in the brain of 106-treated YG8R mice are shown compared with vehicle-treated controls set at 100% (*n* = 4). Error bars represent s.e.m.

## Conflict of interest statement

Repligen Corporation has acquired a license from Scripps Research Institute for the development of the pimelic *o*-aminobenzamide HDAC inhibitors first synthesized in the lab of JMG. Repligen filed for patenting of the molecules described in this manuscript. GB, SJ and JRR are employees of Repligen and JMG is a consultant for Repligen. This corporate involvement does not alter the authors' adherence to all of this journal's policies on sharing data and materials.
