# Supplementary figures and images for: Prolonged treatment with pimelic o-aminobenzamide HDAC inhibitors ameliorates the disease phenotype of a Friedreich ataxia mouse model
Source: Neurobiol Dis. 2011 Jun;42(3):496–505. doi: 10.1016/j.nbd.2011.02.016 (PMC3107941; doi:10.1016/j.nbd.2011.02.016)

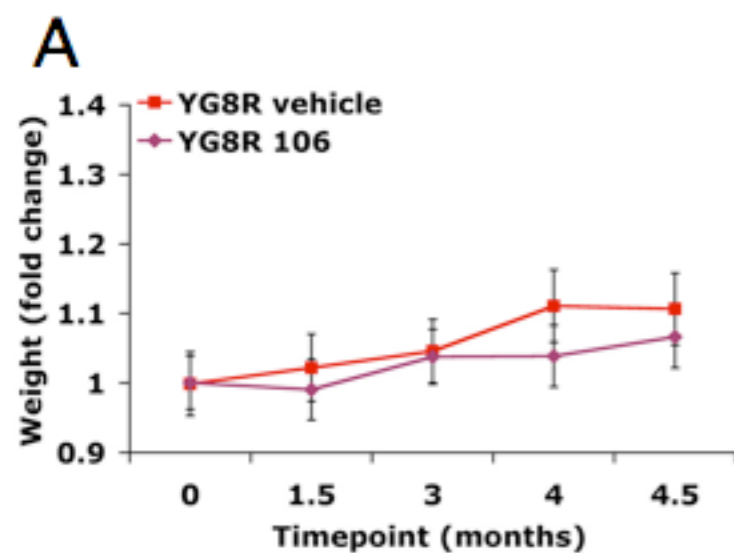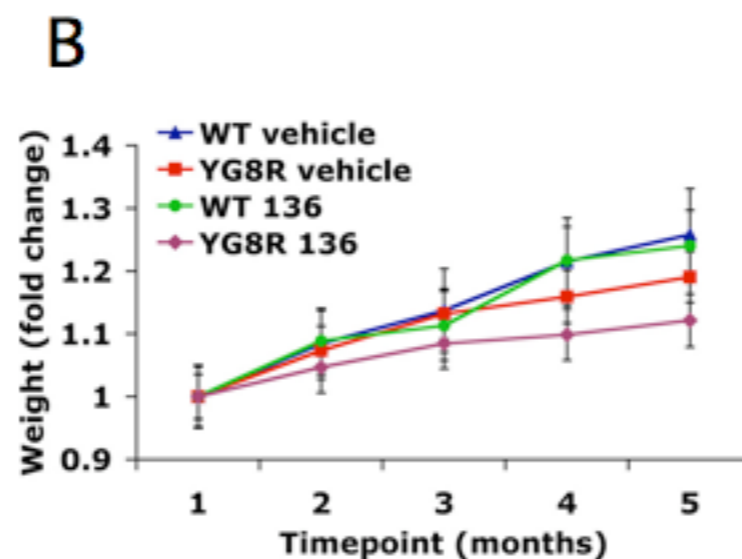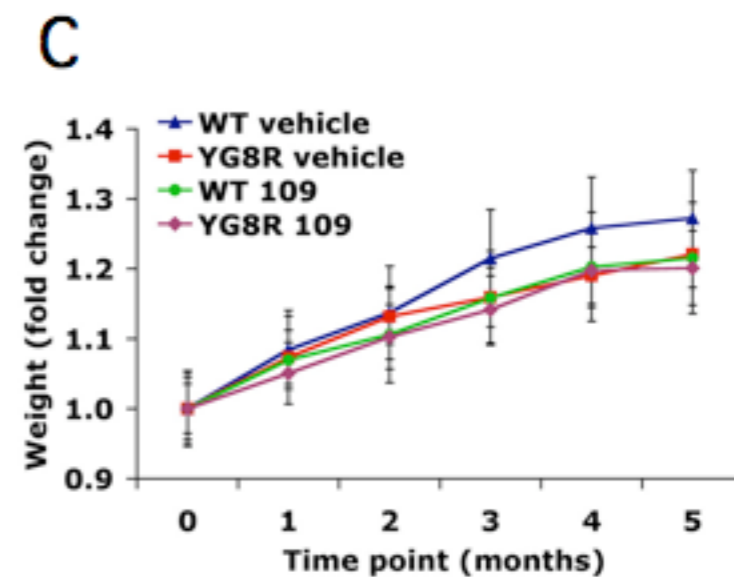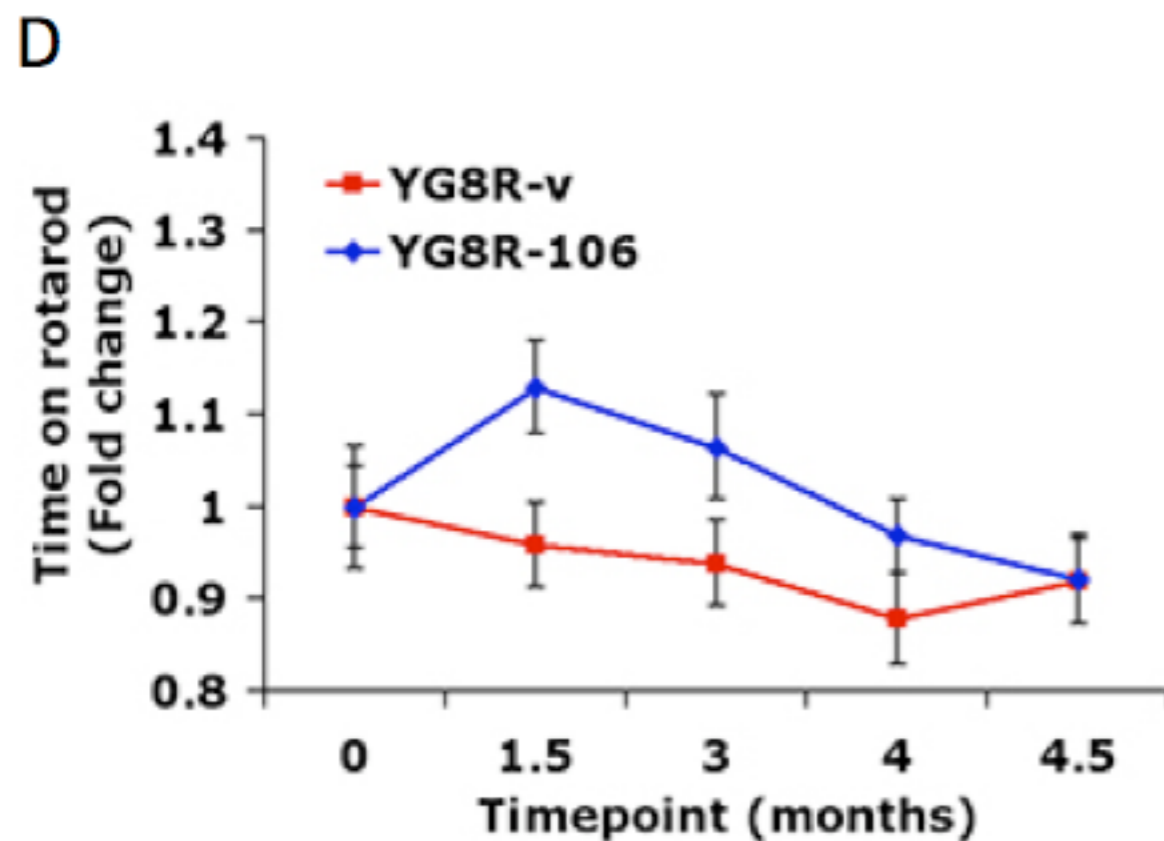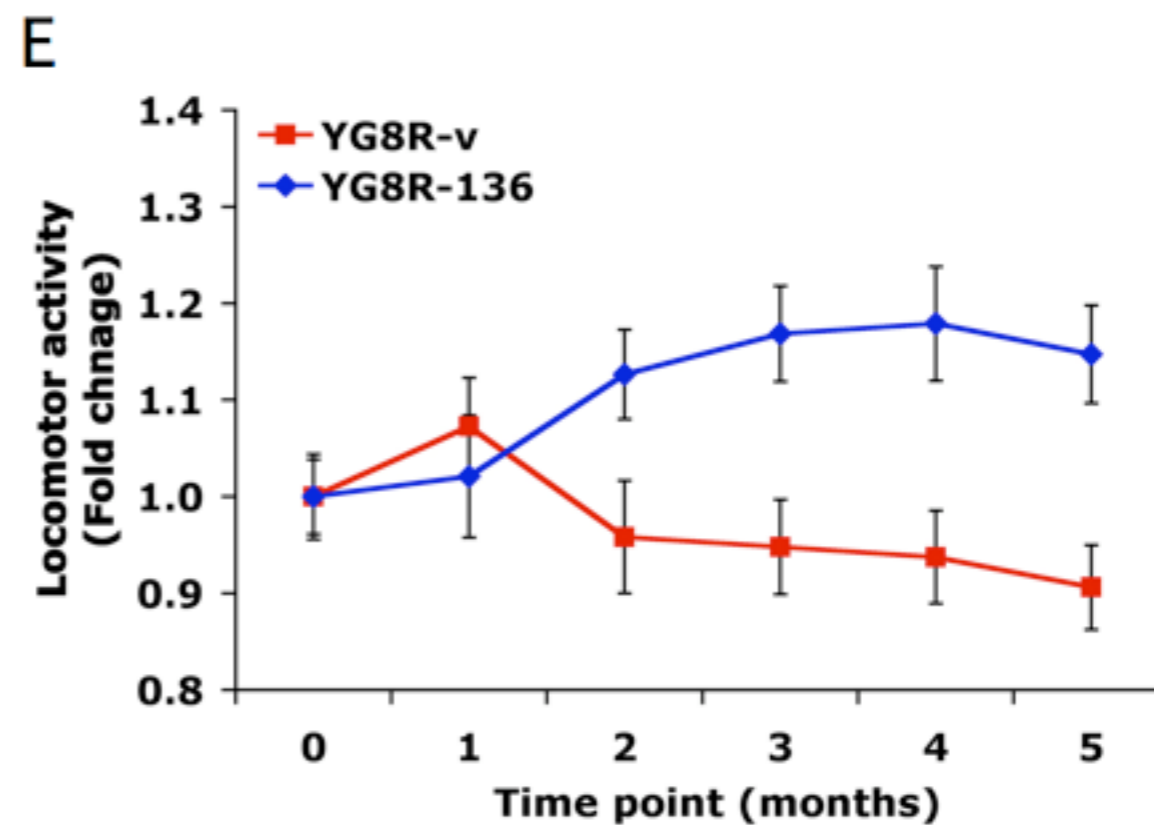

Supplement: Fig. S1 — Weight and behavioral analysis. (A–C) Fold changes in body weights are shown for vehicle-treated YG8R (red) and wild-type (WT) (blue) mice and HDAC inhibitor-treated YG8R (purple) and wild-type (WT) (green) mice over a 5-month time period. (A) 106 (n = 15), (B) 136 (YG8R n = 20, WT n = 10) and (C) 109 (YG8R n = 20, WT n = 10). (D) Relative rotarod performances of 106-treated (blue) and vehicle-treated (red) YG8R FRDA mice (n = 15). (E) Relative open field locomotor activities of 136-treated (blue) and vehicle-treated (red) groups of YG8R FRDA mice (n = 20). Error bars represent s.e.m. [file mmc1.pdf]

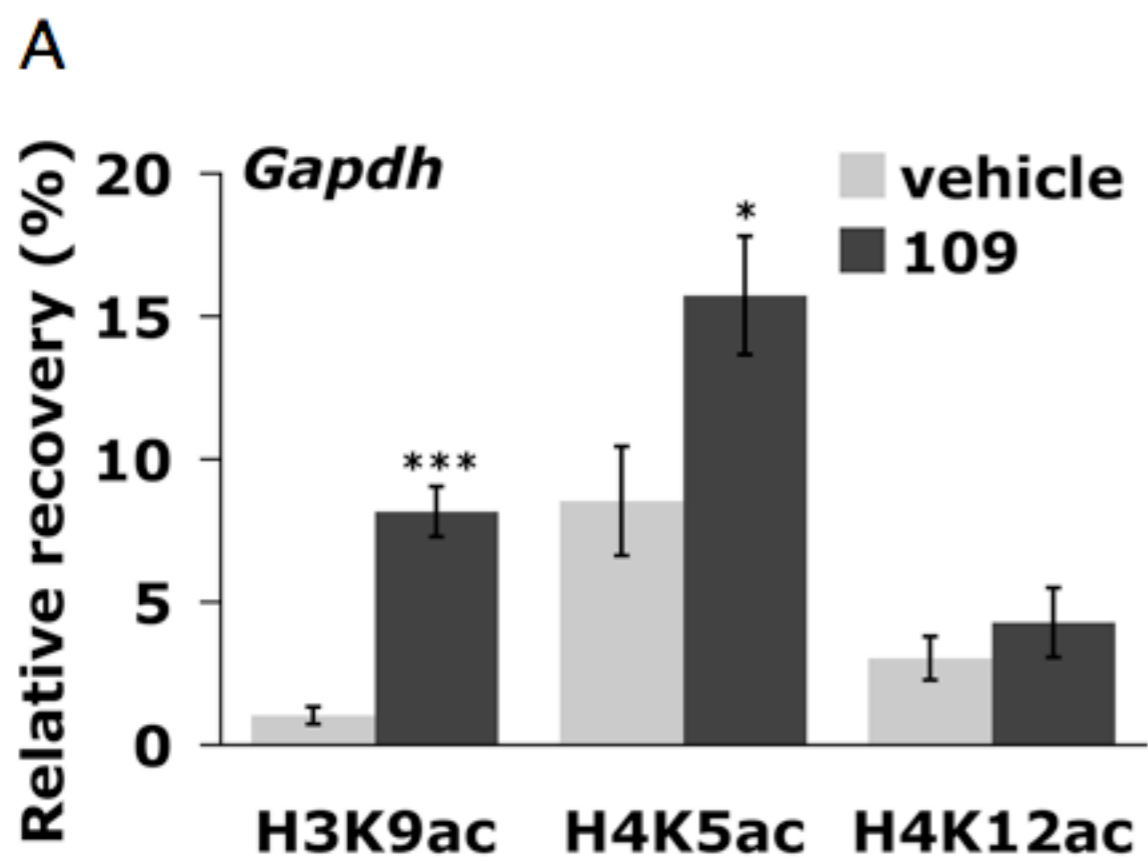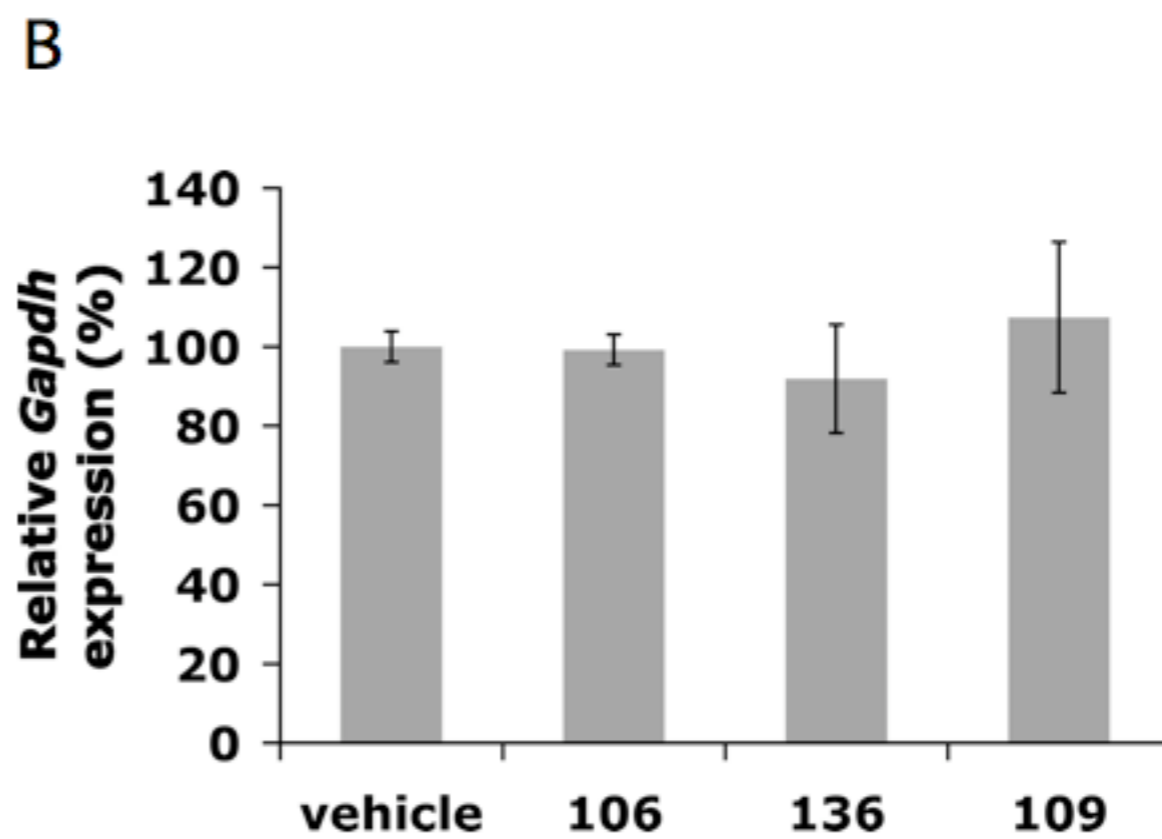

Supplement: Fig. S2 — Gapdh ChIP and mRNA expression levels. (A) ChIP analysis of acetylated H3K9, H4K5 and H4K12 residues within the endogenous mouse Gapdh gene of 109- and vehicle-treated YG8R mouse brain tissues are represented as relative recovery (%), which was calculated as the amount of immunoprecipitated DNA compared with input DNA, subtracting the –Ab background value (n = 6). (B) Relative Gapdh mRNA expression of YG8R mouse brain tissue, compared with a vehicle-treated control value set at 100% (n = 3). Error bars represent s.e.m. *p < 0.05, ***p < 0.001. [file mmc2.pdf]

**A**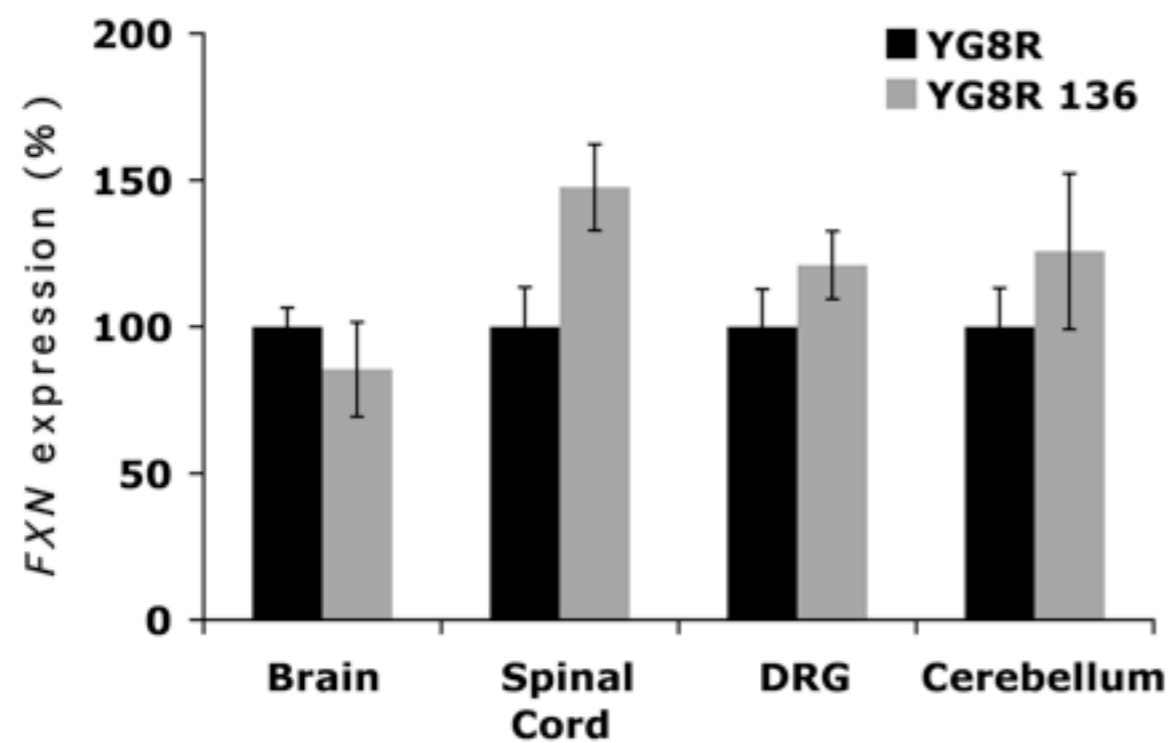**B**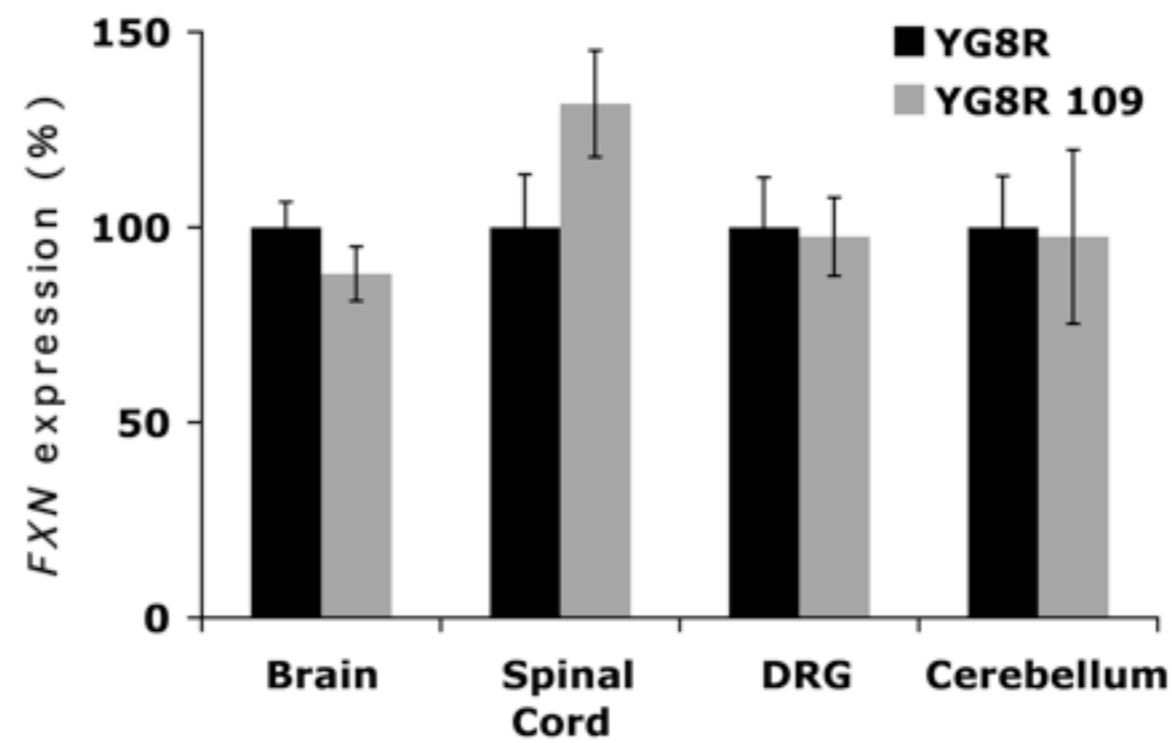**C**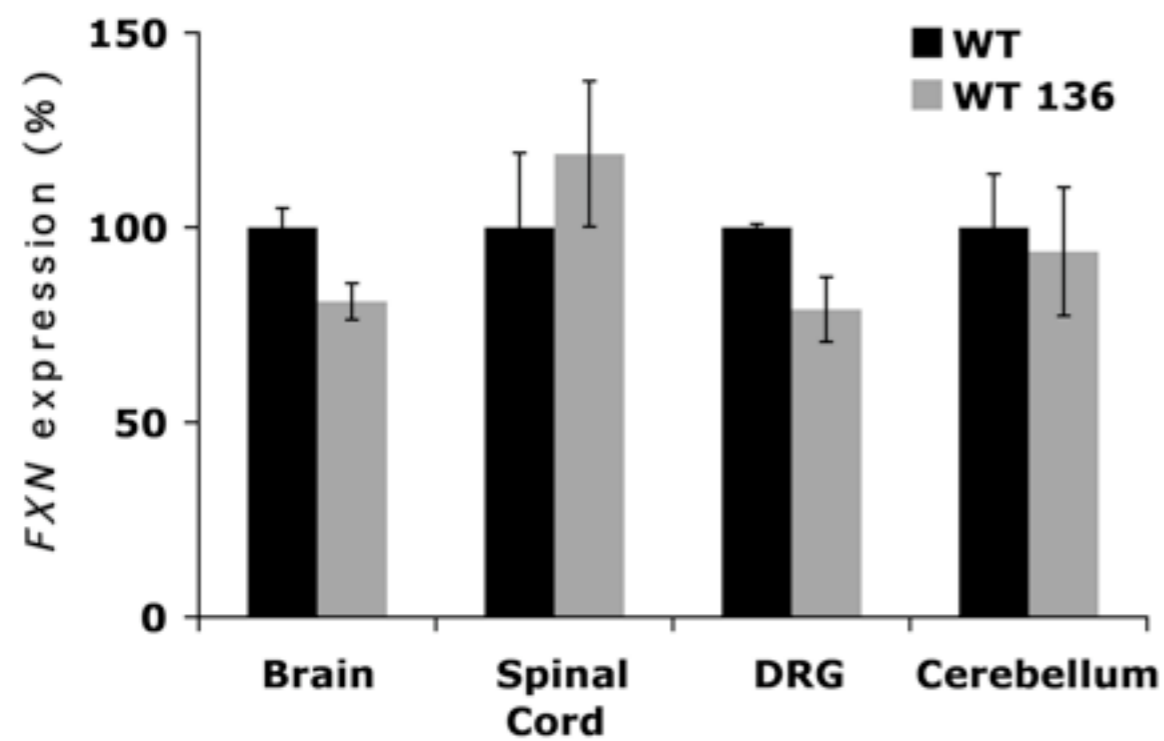**D**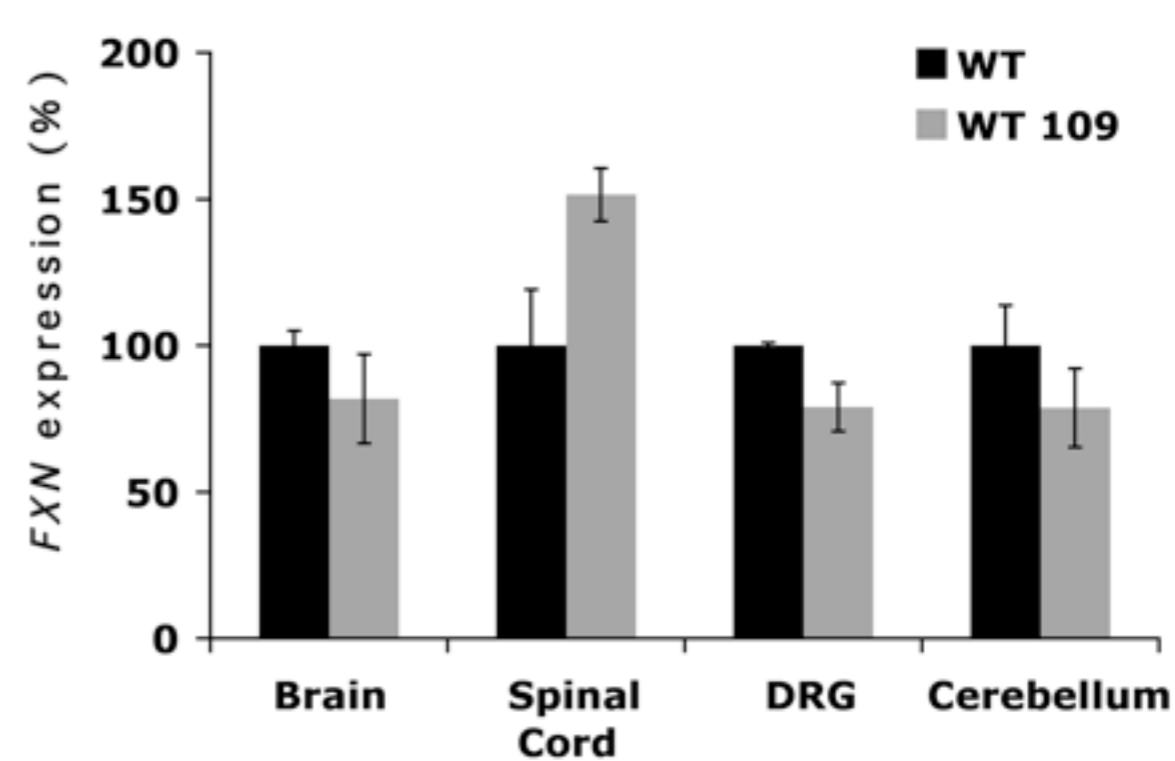

Supplement: Fig. S3 — Relative FXN mRNA levels in HDAC inhibitor-treated YG8R and wild-type brain tissues. Relative FXN mRNA expression levels in the brain, spinal cord, DRG and cerebellum of YG8R mice (A, B) and wild-type mice (C, D) are shown after treatment with 136 (A and C) and 109 (B and D), compared with corresponding vehicle-treated controls set at 100%. In all cases n = 4. [file mmc3.pdf]

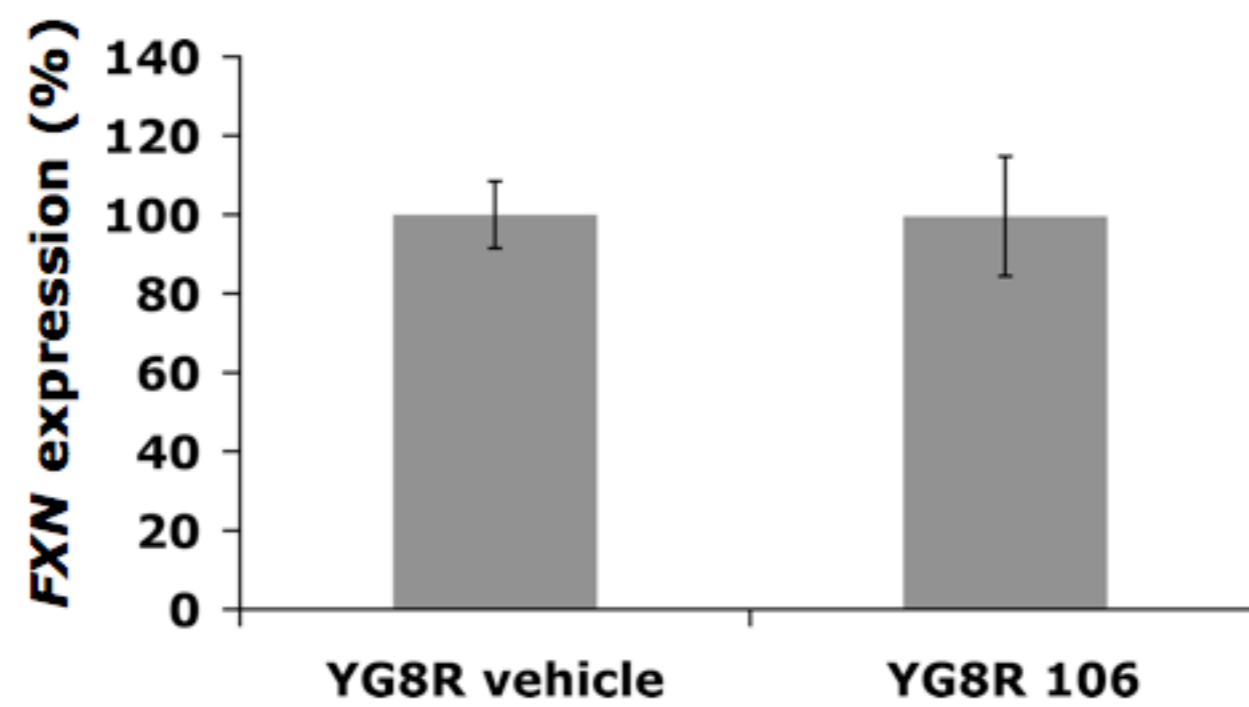

Supplement: Fig. S4 — FXN mRNA levels in 106-treated brain. Relative FXN mRNA expression levels in the brain of 106-treated YG8R mice are shown compared with vehicle-treated controls set at 100% (n = 4). Error bars represent s.e.m. [file mmc4.pdf]
